# Functional Neuroimaging in Traumatic Brain Injury: From Nodes to Networks

**DOI:** 10.3389/fneur.2017.00407

**Published:** 2017-08-24

**Authors:** John D. Medaglia

**Affiliations:** ^1^Department of Psychology, University of Pennsylvania, Philadelphia, PA, United States

**Keywords:** connectome, TBI, graph theory, fMRI, brain reorganization

## Abstract

Since the invention of functional magnetic resonance imaging (fMRI), thousands of studies in healthy and clinical samples have enlightened our understanding of the organization of cognition in the human brain and neuroplastic changes following brain disease and injury. Increasingly, studies involve analyses rooted in complex systems theory and analysis applied to clinical samples. Given the complexity in available approaches, concise descriptions of the theoretical motivation of network techniques and their relationship to traditional approaches and theory are necessary. To this end, this review concerns the use of fMRI to understand basic cognitive function and dysfunction in the human brain scaling from emphasis on basic units (or “nodes”) in the brain to interactions within and between brain networks. First, major themes and theoretical issues in the scientific study of the injured brain are introduced to contextualize these analyses, particularly concerning functional “brain reorganization.” Then, analytic approaches ranging from the voxel level to the systems level using graph theory and related approaches are reviewed as complementary approaches to examine neurocognitive processes following TBI. Next, some major findings relevant to functional reorganization hypotheses are discussed. Finally, major open issues in functional network analyses in neurotrauma are discussed in theoretical, analytic, and translational terms.

## 1 Introduction

What can functional neuroimaging tell us about the injured brain? Quite a lot. There has been explosive growth in functional neuroimaging technology and methods since the invention of blood oxygen level-dependent functional magnetic resonance imaging (BOLD fMRI (1)). fMRI allows us to examine cognitive resilience and dysfunction, changes in brain dynamics, and neuroplasticity following brain trauma. With ongoing developments in the field, it is useful to identify theoretical frameworks that concisely integrate findings and produce testable hypotheses.

Here, I describe past, current, and future directions in fMRI research applied in traumatic brain injury (TBI) within an integrating perspective known as “cognitive network neuroscience” (2). In this theoretical framework, cognitive function depends on time-evolving (3, 4), multiscale (5), heterarchical (6) processes in brain networks. Traditional neuroimaging combined with modern tools from *network science* allows researchers to investigate the nature of cognitive function within brain networks, how networks and cognition are disrupted by brain trauma, and how they change over time following injury. To achieve this, I review three core areas for fMRI research in TBI: how to understand BOLD fMRI signals in the injured brain, major analytic approaches applied to fMRI data in TBI, and important frontiers to achieve a mature network science in fMRI research applied to TBI. First, some observations about the nature of TBI in the population and its pathophysiological effects provide a broader context for this effort.

## 2 The TBI Epidemic and Basic Pathophysiology

As context to understand any functional changes following TBI, a brief overview of the nature of TBI and its pathophysiological effects is essential. Closed TBI can result from events that cause the brain to move rapidly within the skull, such as impacts, blast waves, and rapid acceleration and deceleration (7). In addition, open or *penetrating* TBI occurs when the dura mater is breached by an external object or bone fragments (7). TBI occurs at an epidemic scale, with over 2.8 million new TBI-related medical visits per year and over 50,000 deaths (8). The high survival rate following TBI indicates that the vast majority of patients live afterward with some degree of permanent cognitive loss (9, 10) and psychiatric disturbance especially marked by depression (11).

Within and between mechanisms of injury, TBI is highly variable and no two cases are identical (12, 13). Moreover, two injuries can appear superficially to be very similar in terms of mechanism of injury and the distribution and severity of damage but be associated with very different outcomes (14–16). Some observations about common TBI pathophysiology provide a context to understand the general anatomical contributions to cognitive phenomena. At a high level of brain organization, closed TBI is frequently associated with direct damage to cell bodies in the gray matter due to *coup* and *contre coup* compression of the cortex during injury (17, 18). In addition, focal and diffuse damage to axons can be observed due to rotational forces that stretch and shear axons (19). Typically, TBI severity is classified using the Glasgow Coma scale into mild, moderate, and severe ranges (20, 21). More severe or repeated mild TBI is associated with greater risk of neurodegenerative disease, such as Alzheimer’s (22), chronic traumatic encephalopathy (23), and Parkinson’s disease (24). While the specific pattern of TBI varies, TBI outcome is highly related to age of injury and initial injury severity: younger individuals with less severe injuries demonstrate the best recovery (25).

Despite vast heterogeneity in TBI profiles, some basic microscopic pathophysiological effects can be observed. In the acute phase post-injury (<1 h), the excitatory neurotransmitter glutamate is released rapidly and disrupts ionic equilibrium at the postsynaptic membranes (26, 27). Extracellular potassium ion levels also increase, potentially secondary to increased neural firing (26, 27) due to excitatory neurotransmitters that scales with injury severity (26, 27). Intracellular calcium ion concentrations increase as early as 6 h after injury, approximating healthy levels between 4 and 7 days after injury. Cognitive deficits in the spatial memory domain have been observed to resolve with calcium renormalization by 30 days post-injury in animal models (28), and faster calcium renormalization has been observed in younger rodents (29).

Finally, research examining glucose metabolism following TBI broadly indicates that TBI is associated with a rapid increase in glucose uptake shortly (<30 min) post-injury in animal models (27) and up to 8 days after severe human head injury (30). Following this period, glucose metabolism decreases from 5 to 14 days post-injury in animal models (31, 32) with greater and longer-lasting depression in the penumbra (33). In animal models, the magnitude and duration of glucose metabolism changes are greater in older rodents (33, 34), suggesting an energy-based mediator for cognitive decline following TBI. Indeed, focal glucose metabolism rates following TBI in the thalamus, brain stem, and cerebellum are positively correlated with consciousness measured by the Glasgow Coma Scale (35).

## 3 Understanding fMRI Findings in TBI

**Figure 1:**
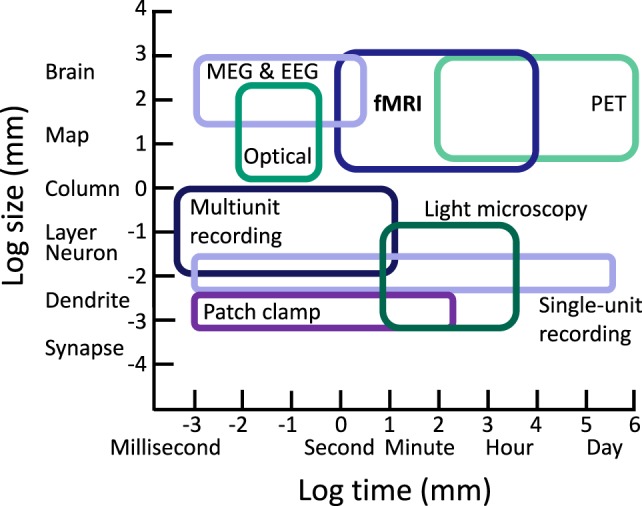
Approximate ranges of spatiotemporal sensitivity of neural measurement techniques. A time by space diagram of the sensitive ranges of various neural recording techniques. fMRI occupies a large space of sensitivity over a scale from seconds to hours and from slightly above the level of neural columns to the entire brain. Importantly, fMRI can measure a range of spatiotemporal organization not accessible to other modern approaches, contributing unique value in characterizing functional changes and testing cognitive hypotheses following TBI. PET, positron emission tomography; MEG, magnetoencephalography; EEG, electroencephalography.

Considered in the context of the complex pathophysiology and variable outcomes observed in TBI, fMRI is one of several tools used to examine functional responses to TBI. However, it is critical to note that while fMRI occupies a sensitive range that is not well sampled by other techniques, it samples only a small portion of the spatiotemporal scale of brain organization and neural activity. To realize a comprehensive picture of the effects of pathophysiology on neural activity following TBI and associated cognitive changes, multimodal approaches are necessary, and we should always be mindful of basic limitations of fMRI when making neurocognitive inferences (see Figure 1).

To review the specific role of fMRI to indirectly examine the neural substrates of cognition following TBI, I first briefly introduce the basis of neural inferences in fMRI data via the hemodynamic response function. Then, I introduce cognitive network neuroscience as a general approach to integrate traditional and network approaches to neuroimaging in cognitive neuroscience. Lastly, I discuss the frequent emphasis on brain reorganization hypotheses in fMRI research and offer operational definitions for fMRI research in TBI that can apply to neurological disorders at large.

### 3.1 Hemodynamics and Neural Dynamics in TBI

**Figure 2:**
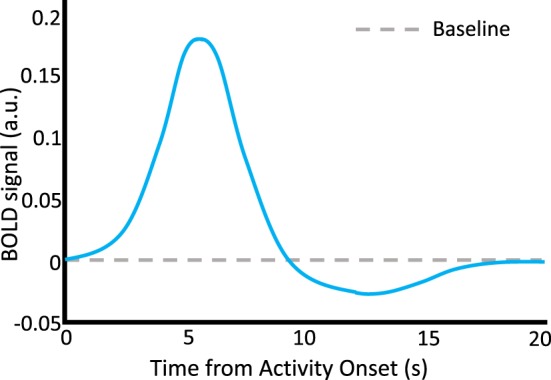
The hemodynamic response function. A schematic representation of the HRF, which describes neurovascular coupling and the basis of the BOLD signal. A discrete episode of neural activity occurs at time = 0 s. A characteristic increase in the BOLD signal occurs with a latency to peak of about 6 s following the neural activity, followed by a decline below baseline between 10 and 15 s, and finally return to baseline by about 20 s. Stimulus time courses in behavioral task designs can be convolved with the HRF to predict time courses of BOLD activity which are thought to represent cognition-relevant neural changes. a.u., arbitrary units.

fMRI analyses rely on our knowledge that there is a relationship between neural activity and the flow of blood to neural tissue. Hemodynamics and neural dynamics are linked through the hemodynamic response function (HRF). The HRF forms the foundation for inferences about neural function in fMRI research in cognitive neuroscience. Researchers capitalize on the fact that the HRF expresses a predictable relationship between neural firing and oxygen intake. A “canonical” HRF model is often used in studies that use general linear models to examine the differences in the BOLD signal as a function of experimental conditions or behavioral measurements. In particular, it is thought that cognitive activity alters the frequency and intensity of neural firing, which in turn modulates the BOLD response in predictable time courses via the HRF (see Figure 2).

In time series analyses where fMRI data are measured as a series of consecutive events, an explicit canonical HRF model may not be used. In this case, we may examine the BOLD fMRI time series in a narrow frequency band that is thought to represent neural firing without contamination by physiological nuisance variables such as respiration or heart rate. In these cases, the time series is thought to represent “spontaneous” neural activity, which can be investigated in paradigms in which subjects are at rest, looking at a fixation cross, or performing cognitive tasks (36).

### 3.2 The Hemodynamic Dilemma

The simple diagram of the HRF above exists in a context of complex relationships between neural activity, the brain, and behavior. While we are often interested in neural responses as the basis of cognition and behavior in health and TBI, the relationship between neural activity and the BOLD response is far from completely understood. Notably, even in health the BOLD response varies substantially across individuals, challenging the widely applied practice of using “canonical” HRFs in general (37). It may be that most of us have the impression that the BOLD signal is a surrogate for action potentials. However, it is not known which specific neural responses do and do not result in a BOLD response across the entire human brain *in vivo*.

The contributions of glia and astrocytes to both cognition and the shape of the HRF are not completely understood. It is not fully known how non-neural physiological effects and regional and individual BOLD variability influence the results presented in thousands of studies since the introduction of fMRI. In light of this, our interpretations based in BOLD imaging should be quite circumspect, and we should continue to examine and clarify what neural activity we are talking about. See Ref. (38) for an excellent review of these issues.

Moreover, TBI represents a special case in which the nature of the injury may fundamentally alter the relationship between neural activity and hemodynamics, thus resulting in a different HRF. If this is the case, the standard challenges to interpreting the BOLD signal are further complicated. Evidence that the HRF could be affected in TBI comes from several sources. TBI has been shown to reduce cerebral perfusion in humans (39), decrease vascular CO_2_ reactivity (40) and decrease both the density and diameters of capillaries at the injury site and diffusely (41). Metabolic failure after TBI can occur in the presence of normal perfusion (42). This involves a decoupling between cerebral blood flow and the cerebral metabolism rate for glucose during baseline states, followed by generally reduced cerebral metabolism (39, 43). Animal models suggest that alterations in cerebral blood flow and the cerebral metabolic rate of glucose are long-lasting physiological effects of concussion (43). The decoupling between cerebral blood flow and the cerebral metabolic rate of glucose in animal models have mostly been observed during anesthesia, when a tight coupling (44) exists between cerebral blood flow and the cerebral metabolic rate of glucose. How these findings generalize to conscious humans during cognition is unknown.

One study applied a simple sensorimotor task in mild TBI *in vivo* in humans and found more spatially distributed and earlier times to peak in the TBI group relative to controls, inferring that additional compensatory neural resources were recruited to support the task in the context of matched performance (45). Unfortunately, this does not directly address the fundamental relationship between local neural firing and hemodynamics following TBI. While we can identify BOLD profiles that may be consistent with cognitive reallocation or latent resources, the full nature of the HRF in TBI remains an area in need of focused research with ground truth data. Our interpretations can be aided by convergent analyses from many perspectives.

### 3.3 Nodes, Networks, and TBI

There are two predominant ways to analyze fMRI data in TBI and studies at large. The first emphasizes BOLD activity *within* regions of the brain, where local changes in BOLD signal amplitude are compared between TBI and a reference (control) group, or related to behavioral or demographic variables. The second emphasizes BOLD activity *between* regions of the brain over times. Either approach can involve analysis conducted on individual voxels in an fMRI image or summaries (e.g., averages) of the activity across many voxels within regions. In principle, there is no limit to the number of analytic techniques that can be used on BOLD fMRI data.

To provide a useful conceptual foundation, a theoretical framework can provide a context in which to understand findings, discriminate among hypotheses, and identify new scientific questions. Human brain networks involve neurons connected in complex patterns that enable cognition. Mathematically, a brain network can be defined as a graph *G* composed of *N* nodes (for current purposes, typically voxels, brain regions) and *E* edges (region-region relationships). In network science, the term *graph* refers to the join-the-dots pattern of connections (edges) between nodes, rather than to a visual representation of data on axes. We examine the pattern of edges linking nodes by quantifying the graph’s structure using a variety of diagnostics, which each provide complementary but not necessarily independent information (46–49). Network representations of complex systems facilitate quantitative analysis of heterogeneous interactions higher order multivariate patterns within a unified mathematical framework (50).

**Figure 3:**
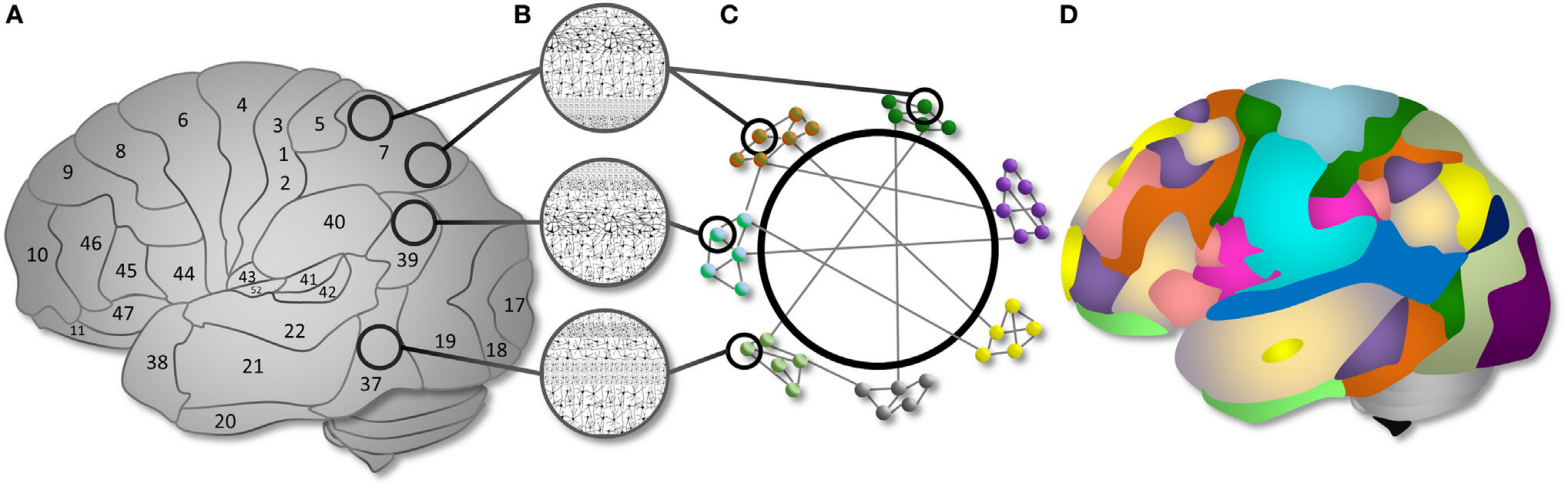
Cognitive network neuroscience as a way to integrate nodes and networks. **(A)** Brain regions are organized into cytoarchitectonically distinct areas. **(B)** Each cytoarchitectural configuration has structural properties with different implications for computational functions. **(C)** Cytoarchitectural regions can be represented as nodes in a network. The nodes have functional associations, represented as edges, that extend beyond spatial boundaries evident in cytoarchitectural organization. Subsystems can be described as network modules. Modules have varying intra-connectivity and inter-module connectivity in the human brain. **(D)** An example topology of the modular organization of functional brain networks demonstrating the communication between computational resources of different types. Distinct brain networks are recruited as “modules” to support specific cognitive functions via within- and between- system interactions. Figure and caption reproduced from Ref. (2) with permission; see the same article for a full exposition.

These advantages are particularly powerful in the study of the human brain. Since 1909, we have known that different brain regions exhibit distinct microanatomical configurations (51), and numerous studies in the past century validate the notion that distinct brain regions support different functions. From network analyses, it is also clear that distinct brain regions are organized into *modules* that communicate over time at rest (52) and are recruited as systems under different cognitive conditions (52, 53). Thus, across levels of brain organization, *cognitive network neuroscience* focuses on complex interactions between spatially discrete brain regions (or “connectome” (54)), represented by graphs, and seeks to link these patterns of interaction to measured behavioral variables (2, 55) (see Figure 3).

In fMRI research in TBI, cognitive network neuroscience offers a perspective that can help integrate across disparate methods and findings. Brain trauma inflicts damage to the brain’s anatomy, which in turn affects its dynamics and associated cognitive function. Thus, a connectomic account of TBI requires study of the local (neuron or region-level) and distributed (functional connectivity and module-level) changes caused by injury, their links with cognitive resilience and deficits, and neuroplastic changes over time following injury. Given the aforementioned complexity in pathophysiology and variability underlying the BOLD response, it is critical that BOLD fMRI studies in TBI anchor analyses to independent biomarkers or measurable behavioral performance to interpret BOLD signal changes. The latter has largely been couched under efforts to understand brain “reorganization,” which I will use to frame current challenges in cognitive network neuroscience applied to study neuroplastic change in TBI.

### 3.4 Brain “Reorganization”

To interpret fMRI signals in the context of brain injury and behavior, a conceptual framework is necessary. In particular, a core notion known as “brain reorganization” has emerged in brain trauma and neurological research. What does “brain reorganization” mean, and how can we identify it when we see it? For a term to be scientifically useful, it must be clearly defined. Here, it is first useful to distinguish *anatomical* from *functional* reorganization. Anatomical organization in TBI refers to the adaptive neuroplastic changes that occur after the primary (e.g., damage to cell bodies and white matter pathways) and secondary (e.g., Wallerian degeneration of axons and local excitotoxic effects) effects of TBI. Gradually, cortical pathway activity expresses excitatory activity accompanied by neuronal proliferation and synaptogenesis. Neurons, endothelial progenitors, glial cells, and inflammatory cells replace damaged cells, promote glial scar tissue, and revascularize (56). In the weeks post-injury, synaptic markers and axonal sprouting are upregulated (57), resulting in neural remodeling that supports recovery. While the distribution of these effects is difficult to examine in humans, comparative work indicates that long-lasting remodeling occurs in the hippocampus after TBI (58, 59). These microcellular changes can be thought to support general structural basis for *de novo* general reorganization following TBI.

Quite distinct from anatomical studies, in the functional neuroimaging literature, “brain reorganization” typically refers to one of two distinct concepts: either (1) the change functional *signals* following brain trauma (such as those quantified with BOLD fMRI or other functional techniques) or (2) when one region obtains a *cognitive* function previously supported by another region following brain trauma. The use of two definitions creates a difficult situation if we do not carefully state the intended meaning of our usage, and indeed leads to challenges in interpreting a broad literature (60). Here, I will discuss how the first usage is scientifically unproductive and potentially misleading. Then, I will suggest how researchers may meaningfully detect the second. For simplicity, I refer to fMRI findings as “BOLD statistics” in the exposition and examples that follow rather than to a specific analytic technique. This is to emphasize that we can apply a potentially universal approach to interpreting BOLD findings in TBI regardless of the statistical analysis we use.

#### 3.4.1 Brain Reorganization As Changes in Functional Signals

Anatomical disruption to the brain alters the hemodynamic and neural processes in surviving tissue. Detecting these changes with fMRI is often referred to as evidence for “brain reorganization,” which is a tautologous expression. To detect whether this meaning is in use, we can check whether the word “change” could replace “reorganization” in a sentence without affecting the meaning of the sentence. For example, if a researcher states something like “We observed increases in prefrontal cortex BOLD amplitude during working memory performance, indicating that the brain reorganized to support task performance,” we could instead state that “the brain changed to support task performance” without any obvious difference in the meaning of the sentence. Here, the use of “reorganization” gives us the false impression that something has been learned *about* the change in the prefrontal cortex, but this cannot occur without a notion for how to assess a mechanism^1^ responsible for the observed BOLD change. Thus, it is preferable simply to describe the observed physiological in terms of the quantitative statistic used to quantify the difference in the TBI sample, as it is important to document physiological changes even when a cognitive interpretation is not facilitated by the data or analysis. If instead a cognitive interpretation is desired, a logical framework expressing the relationship between the brain signal and behavior in principle is important to clarify. Then, we can explicitly test this relationship, anchoring BOLD signals to an outside measure to lend interpretive value.

#### 3.4.2 Brain Reorganization As Reallocated Cognitive Mechanisms

The second meaning of “brain reorganization” is an interesting area of research that can be grounded in a cognitive framework. Neuroplastic changes that reallocate cognitive functions in the brain are among the most important concepts in clinical cognitive neuroscience. If we can design studies that detect this type of “brain reorganization,” we can advance scientific knowledge that elucidates why some cognitive functions are more vulnerable than others and how to produce therapies that capitalize on models of cognition-relevant neuroplastic changes. For clarity, I refer to this meaning of brain reorganization as “cognitive reallocation.” To identify reallocated cognitive mechanisms, it is necessary to clarify the cognitive framework and how it relates to statistical analyses. As a case to serve the point, let us imagine that, relative to controls, we observe a new set of BOLD signals in a damaged brain during a cognitive function, such as altered amplitudes, connectivity, or a time-varying network pattern. In the context of brain reorganization, an important starting point is to ask “how can we know when a brain region obtains the function of another region?”

There are reasonable starting points to answer this question. If the functional activity of a brain region *A* has a consistent BOLD response profile in some cognitive conditions but not others, we can construct experiments that detect its response and clarify its mechanistic role. Indeed, this is a foundation for fMRI research in cognitive neuroscience. As a simple example, assume that region *A* is necessary for working memory load maintenance. Now, imagine that a brain trauma destroys region *A*. By definition, a missing region cannot perform the function that it once performed. However, imagine that we obtain convincing behavioral evidence that working memory load maintenance, at least in some form, persists despite the destroyed region. Logically, either the assumption of necessity was incorrect, or some other brain region *B* now performs the function *A* once performed to support working memory maintenance.

**Figure 4:**
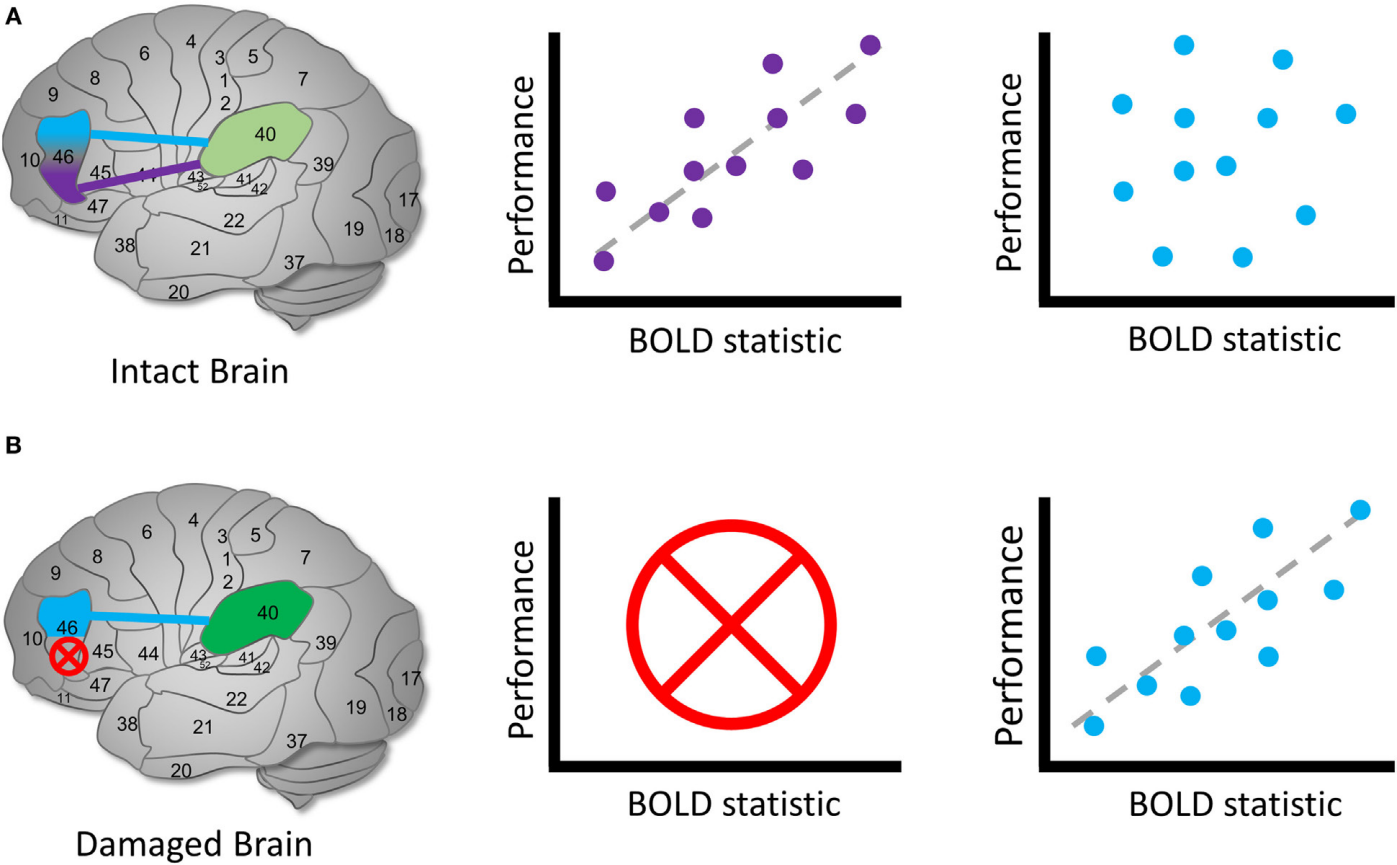
Identifying reallocated cognitive function. Hypothetical diagram illustrating how to identify candidates for a brain reorganization represents reassigned cognitive function. **(A)** In an intact brain, we might observe that a brain region can be functionally dissociated into two parts. *Left*: Brodmann’s area 46 separates into two regions that communicate with area 40. *Middle*: the purple region’s BOLD signal or functional connection exhibit a measurable relationship with performance during a cognitive task, whereas *(right)* the blue region’s signal or connection does not. In this case, we identify the purple region to be involved in the cognitive process of interest in health. **(B)** In a damaged brain, *(left)* the original purple region is destroyed entirely and *(middle)* can no longer contribute a cognitive function. *Right: we* observe that the blue region’s signals or functional connections now occur in a similar pattern to the purple region in the healthy sample. We can identify the blue region as a possible site of a reassigned mechanism. The schematic of the relationship between brain signals and performance is arbitrary; the direction and shape of the relationship between the BOLD statistic and performance may vary across the brain and cognitive domain (64).

How can we find region *B*? Knowing nothing else, we should aim to detect a new BOLD signal profile similar to *A* in all respects.^2^ In this best-case scenario, we perform the same experimental design to elicit the same BOLD profile as *A* and detect its spatiotemporal signature. If we successfully identify such a signature in the same conditions, it is a reasonable candidate for *B* (see Figure 4). In general, we often have prior hypotheses about the possible candidates for region *B*, such as the idea that homotopic brain regions are the most likely to obtain the reorganized function (63). Note that this same logical approach can apply to regions as well as functional connections among regions, entire circuits or systems, or any other brain quality we can use fMRI to investigate.

This account relies on a key idea: understanding how a system normally works is often important to interpret changes when it is damaged. This framework also connects the study of brain reorganization using BOLD fMRI to the essential logic of necessity and sufficiency, double dissociations (65), and forward inference in cognitive neuroscience (66). The logic of cognitive neuroscience research design and hypothesis testing can form a strong framework to clarify the nature of cognitive reallocation in neurological research.

#### 3.4.3 The Perils of Brain Reorganization

If brain reorganization can be synonymous with both “change” in brain signals and “cognitive reallocation,” confusion may ensue. There are two potential ways to maintain clarity. The first is that we can be very clear to indicate what we mean by “brain reorganization” by stating it explicitly in writing and conversation. The second is to replace it altogether with more specific and meaningful terminology. If it is reasonable to do the former, then it may be preferable to do the latter in every case. If we intend “brain reorganization” to refer to a change in brain signals, then it is always clearer to refer to the signals by name, whether they are BOLD signal amplitudes or measures of functional connectivity. If we intend “brain reorganization” to mean “reallocated cognitive mechanism,” then it is more precise to state the hypothetical signals associated with the cognitive function and create research designs to identify them. In either case, “brain reorganization” serves as a vague proxy for a more meaningful term. Thus, we should be circumspect in using the term and consider dropping it altogether to the benefit of scientific discourse.

### 3.5 Compensation and Latent Mechanisms

Once we identify a candidate for a reallocated cognitive mechanism, it is critical to dissociate cognitive reallocation from other sources of BOLD responses following TBI. This distinction is important because if the brain has two types of response to injuries, it is possible that the mechanisms governing these responses operate differently, with different practical and translational consequences. For example, if we aim to create smart therapies that elicit specific types of neuroplastic changes, we would not want to induce a cognitive reallocation if instead another process was at work. At best, the therapy could fail, and at worst, we could interfere with another intact process.

**Figure 5:**
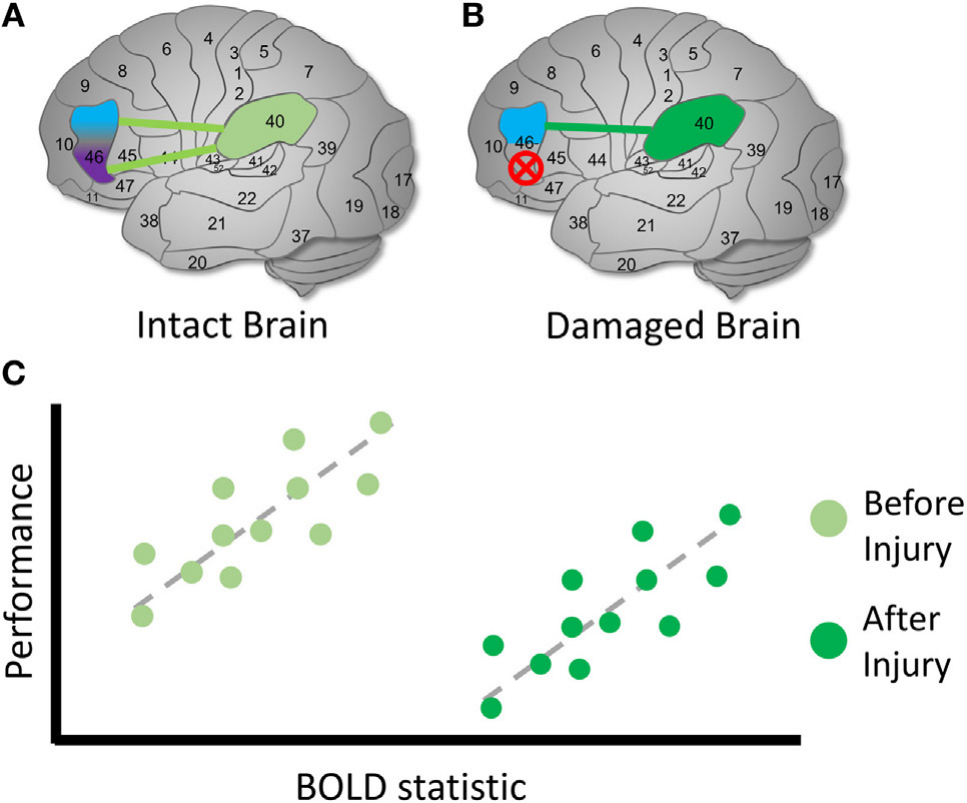
Identifying compensation following TBI. Hypothetical diagram illustrating how to identify possible latent compensatory mechanisms. **(A)** In an intact brain, we observe the same initial relationship described in Figure 4. However, suppose that we are interested in whether activity involving BA 40 may represent a compensatory mechanism following TBI. **(B)** In a damaged brain, the original purple region is again destroyed entirely and can no longer possibly contribute to the cognitive function. **(C)** We examine the relationship between BOLD signals involving BA 40 and measures on cognitive tasks. In the intact brain, unlike a reassigned cognitive function, a relationship is observed between the BOLD signal and BA 40. Following TBI, we observe the same relationship between the BOLD statistic and performance, but the mean BOLD statistic value is higher following injury, and mean performance is lower. This is one possible sign that a compensatory latent mechanism is recruited in BA 40 [see also Ref. (60, 67, 68)]. The higher mean BOLD statistic may make it easier to detect in the TBI sample, suggesting that thresholding is an important decision point in this case. As in the previous example, the direction and shape of the relationship between the BOLD statistic and performance may vary across the brain and cognitive conditions.

Two other bases for altered functional brain responses following TBI include *compensation* and *latent resources/mechanisms* (67, 68). Compensation typically refers to a cognitive function that is brought online following brain injury in reaction to the loss of a different function. Distinct from cognitive reallocation, compensation involves mechanisms that existed before the injury but were not relied on as heavily. For example, if a patient suffers from fluency impairments due to frontal lobe damage, we might hypothesize that they compensate by using non-verbal strategies represented by signal recruitment in visuospatial processing systems. In another scenario, an individual with diffuse axonal injury may rely heavily on cognitive control mechanisms to maintain items in memory or execute plans. This latter phenomenon is expressed in increased BOLD amplitude in fronto-parietal control systems after closed brain trauma (60, 68) and will serve as an example henceforth. As in cognitive control in TBI, there are some scenarios where it is possible to detect a compensatory response that is distinct from cognitive reassignment based on the nature of the brain region’s BOLD response profile in health compared to after TBI. Importantly, a compensatory response occurs in a brain region that is intact both in health and following TBI (see Figure 5).

Distinguishing a latent compensatory mechanism from a reassigned cognitive mechanism is challenging in certain cases. Specifically, if a compensatory mechanism does not exhibit a BOLD–behavior relationship before brain injury but does after injury, it may be indistinguishable from cognitive reassignment. In the preceding example, if BOLD activity involving BA 40 id not relate to performance before injury but does afterward, we might conclude that cognitive reassignment from BA 46 to BA 40 has occurred. However, it is also possible that BA 40 contains distinct mechanisms that are not normally associated with performance in health, but activated in the context of brain injury (e.g., the recruitment of spatial representations or attention control processes). In this case, it is important to test multiple dimensions of cognitive function: if BA 40 exhibits a BOLD profile that looks similar to pre-injury BA 46 BOLD activity on all accounts, it may represent reassigned cognitive function. However, if BA 40 exhibits a BOLD profile consistent with cognitive functions across multiple experimental conditions that are not consistent with the healthy BA 46 BOLD profile, it increases confidence that its post-TBI activity represents a compensatory response. This reinforces that regardless of the power of a statistical technique, sound experimental design is a prerequisite to interpreting BOLD signals in the context of TBI and neuropathological syndromes at large. fMRI is a tool that can support this effort by facilitating numerous types of functional analysis in the human brain.

## 4 fMRI: Techniques to Study the Damaged Brain

Once we have acquired BOLD data, we must select tools for analysis to facilitate inferences. There is no limit to the tools we can apply to analyze BOLD signals in health and disease. In principle, any spatio-temporal data analysis technique can be used if the technique’s assumptions are met. This emphasizes the opportunities as well as challenges in TBI research: on the one hand, we can discover important facets of complex spatiotemporal BOLD changes in TBI in the context of behavior. On the other hand, we risk difficulty in interpreting findings given that the options are endless. The relatively simple framework of definitions and hypothesis testing described above can apply to a wide range of measured BOLD phenomena in TBI. To proceed productively, we can select analytic tools that address specific questions, and update our theoretical models of cognitive and neuroplasticity in TBI in light of new findings. First, I briefly introduce “preprocessing steps” used on data prior to primary statistical analysis. Then, I describe the basis of statistical techniques and their utility in fMRI research in TBI, and refer the reader to useful primary resources for each analysis.

### 4.1 Preprocessing

Prior to statistical analysis, we use “preprocessing” techniques to ensure that data meet several assumptions prior to analysis. We aim to minimize the influence of physiological and data acquisition artifacts (such as time lags introduced across brain slices), standardize brain region locations across subjects, and reduce motion effects. The most standard steps to preprocessing fMRI data include slice timing correction (69), motion correction ((70), see also (71) for special considerations in functional connectivity analyses), realignment (72), coregistration of anatomical and functional images (73), spatial normalization (74), and smoothing (75). This final step increases signal to noise, normalizing error distributions, and accommodates anatomical and functional variation between subjects. Most parametric tests assume normal error distribution; and according to the central limit theorem, the distribution of an average tends to be normal with a sufficiently large number of independent observations (76).

### 4.2 Statistical Techniques for fMRI in TBI

Several popular methods more generally applied to fMRI data can elucidate the effects of TBI on the human functional connectome. Many techniques in each analysis class can be productively applied to fMRI data to characterize BOLD signal properties and relate them meaningfully to behavior. These include the general linear model and pattern analysis, other multivariate time series analysis techniques that examine associations among nodes in time series, matrix factorization and decomposition techniques, and network analyses based in graph theory.

#### 4.2.1 The General Linear Model and Pattern Analysis

The general linear model (GLM) refers to a statistical linear model that encompasses statistical techniques, including analysis of variance (ANOVA), analysis of covariance (ANCOVA), multivariate analysis of variance (MANOVA), multivariate analysis of covariance (MANCOVA), linear regression, t-tests, and the F-test. In the context of fMRI, the independent variables often represent behavioral measurements or experimental conditions, and the dependent variables may represent any type of BOLD measurement (or vice versa). BOLD measurements often include signal amplitude or measures of functional connectivity at the voxel or region level.

GLM applied to BOLD fMRI data allows us to apply a relatively simple and statistically well-defined class of tools to examine associations between BOLD characteristics and behavior. In BOLD fMRI research, the GLM combined with a theory of Gaussian fields is known as “statistical parametric mapping” (SPM) (77, 78). This framework facilitates comparisons of BOLD signals across groups, over time, and as a function of demographics or performance on tasks performed in or out of the scanner. As such, it forms the backbone of tools we can use to test for differences and associations, and if a temporal dimension is available in a longitudinal design, prediction. Thus, the GLM is a robust and useful approach in fMRI research in TBI. Using the GLM, we can pose questions to discriminate among competing accounts of brain function and change following TBI (60, 67, 68). We should select a GLM analysis when examining a hypothesis following TBI when we are especially interested in voxel-level differences between groups or relationships with behavior cross sectionally or over time. This allows us to examine region-level hypotheses commensurate with approaches used since the inception of fMRI BOLD contrast imaging.

Extensions of the GLM and other parameter estimation techniques can be coupled with sophisticated machine learning approaches to conduct multivariate pattern analysis for fMRI data. If this approach is used on the voxel level, it is known as “multivoxel pattern analysis” (MVPA) in cognitive neuroscience (79). In addition, pattern analysis can be applied to patterns of functional connections to classify groups (80) and predict behavioral performance at the trial level (81, 82). If we anticipate that unique predictive value is represented across voxels, regions, or functional connectivity profiles, multivariate pattern analyses can provide an important tool to quantify predictors in TBI. We should select a multivariate pattern analysis approach if we anticipate that the specific pattern of activity in one or more regions in the brain is associated with an important group or cognitive information. The unique value of the multivariate approach can be tested against more traditional GLM measures such as mean univariate voxel activity or connectivity. For example, if we anticipate that TBI distorts the information representation in a particular region, we can examine whether the multivoxel pattern in the region accurately identifies TBI subjects relative to controls, and importantly whether behavioral deficits or resilience can be associated with a specific pattern. In principle, the GLM and multivariate pattern analysis can be applied to examine the parameter distributions and network profiles reviewed in the next sections.

#### 4.2.2 Other Multivariate Time Series Analysis Techniques

While GLM approaches in fMRI typically examine the relationships between BOLD measurements, demographics, behavior, or group assignments, we may also be interested in functional relationships among nodes (the so-called “functional connectivity” analyses) as the focus of study. These approaches form one type of brain *network* analyses. These techniques can be applied to time series extracted from individual voxels or regions comprising several voxels. Each technique expresses the relationships among the time series with a distinct emphasis on the objective for the analysis.

In one influential study investigating the performance of connectivity analyses *in silico* based on realistic simulated fMRI data, connectivity models that involve a time lag (i.e., Granger Causality implemented with multivariate vector autoregression), higher ordered statistics, and directionality performed poorly, whereas correlation-based techniques were successful in recovering known simulated model parameters (83). For brevity, I briefly introduce the best-performing techniques tested in this simulation study as well as additional recently validated technique that successfully recover time lags among fMRI series and the *dynamic causal model*.

Full correlation analyses in fMRI require computing the covariance among BOLD time series normalized to unit variance between pairs of regions over time. Full correlation analyses on bandpass filtered data typically performs less well than filtered data, with connection sensitivity becoming poorer in higher frequencies. *Partial* correlation analyses refer to normalized correlation between pairs of time series after each has been adjusted by regressing out all other time series in the data (i.e., the time series of other network nodes). This attempts to distinguish direct from indirect connections. Partial correlation can be considered a surrogate for structural equation modeling (SEM) in the sense that SEM parameter estimation is driven by the orthogonal portions of any given regressor in the model (83). The elements of partial correlation matrices can be compared by standardizing correlation coefficients with Fisher’s R-to-Z transformation (84). We can select a correlation analysis when we have no specific interest in the time-lagged associations between time series in the brain. The correlation matrix between regions can be compared between groups and the correlations between regions can be associated with behavior to test hypotheses about connectivity–behavior relationships.

Inverse covariance (ICOV) analysis is an efficient way to estimate a full set of partial correlations in short fMRI scanning sessions, where we can use regularization parameters to introduce sparsity in inverse covariance matrices. ICOV is a slightly more “model-based” approach than partial correlation because it involves a regularization parameter in its estimation. Using L1 precision to implement ICOV, the regularization parameter *λ* has been found to provide the best results at values of 5 and 100 (83). ICOV without the use of a regularization parameter gives the same results as partial correlation. We can apply ICOV analyses when we have similar hypothetical interests that we would examine in standard correlation analyses but when we are additionally interested in controlling the sparsity of connectivity.

SEM is widely applied in statistical analysis at large and the GLM can be thought of as a special case of SEM. SEM carries the same assumptions as the GLM. In fMRI, traditional SEM is limited because it does not include lagged (autoregressive) effects, which are known to exist in fMRI time series (83). On the other hand, analyzing lagged effects without estimating contemporaneous effects can lead to biased lag estimates. To address this, Kim and colleagues developed the *unified* SEM (uSEM) for use in fMRI data to simultaneously estimate the contemporaneous and time-lagged effects (85). This represents an “effective” connectivity technique in which directed connections are estimated in both the contemporaneous and time-lagged portions. The *extended* uSEM (euSEM) includes an important extension: the effects of input, which can represent task conditions or behavior explicitly in the model. The uSEM and euSEM perform well within an estimation technique known as “group iterative multiple model estimation” (GIMME), which identifies reliable and valid group and individual connectivity structures even when data are highly heterogeneous across individuals comprising the group (86). We can select uSEM and its variants when we are interested in explicitly modeling and testing differences in lagged and contemporaneous associations between a limited number of regions in the context of one another. Because time lags are expected as cognitive processes propagate through the brain, testing lagged associations between regions on the time order of seconds might provide information about processing speed delays in TBI.

Finally, the dynamic causal model (DCM) (87) is a technique that incorporates a biophysical model. It uses the “Balloon model” (88) that describes the transformation of neural activity into a BOLD response. With this model, the DCM estimates the latent neural state space presumed to generate the observed BOLD fMRI time series. Thus, its use of the Balloon model facilitates a neural interpretation under the constraints of the model’s assumptions. Dynamic causal models involve stochastic or ordinary differential equations (i.e., continuous time non-linear state-space models). These equations model the dynamics of hidden states in the nodes of a network, where inter-node dependencies are represented as directed effective connectivity. DCM was originally developed to estimate coupling among brain regions and how it is influenced by experimental changes, and has additionally been extended to apply to resting state fMRI data with stochastic (89) and time-lagged effects (90). We can select a DCM analysis in similar circumstances to the uSEM where a biologically based model is of interest.

#### 4.2.3 Matrix Factorization and Decomposition Techniques

Additional time series analysis techniques that assess functional connectivity include those that seek basis vectors that the observed data are projected against. The most widely used techniques are factor analysis (FA), principal components analysis (PCA), and independent components analysis (ICA). Each of these techniques represents the observed data *X*, where *X* is a node-by-observation matrix, as a weighted linear combination of the original values in *X* to represent new extracted signals *Y*. The differences among FA, PCA, and ICA are found in the criteria for defining the basis vectors. In both FA and PCA, we use a second-order criterion (covariance reduction) whereas ICA uses a fourth-order criterion (maximizing the absolute value of normalized kurtosis).

While FA and PCA are both based on second-order statistics, FA establishes a formal model predicting observed variables from theoretical latent factors. The factors are linear combinations that maximize the shared portion of variance among the initial variables, representing “latent constructs.” FA uses a variety of optimization techniques to identify the common factor structure, and the result depends on the optimization routine used and starting points for the routines. There is not a single unique solution in FA. FA can be applied in either an exploratory or confirmatory fashion to either blindly identify latent factors or test a hypothesized factor structure. FA assumes that the sample is homogeneous, sample sizes are relatively large (greater than 200 or 5 observations per variable is often used as a rule of thumb), multivariate normally distributed data, linear relationships between variables, and moderate collinearity between variables. See Ref. (91) for a thorough resource.

In PCA, we find basis vectors that explain the highest proportion of variance in the data. The highest ranked basis vector is that which best fits all of the variance in the data. The second basis vector is that which also has this criterion, but must be orthogonal to the first, and so on until as many basis vectors are extracted as the original number of nodes. In PCA, the basis vectors are the eigenvectors of the data’s covariance matrix. After PCA, the principal components have 0 covariance between them, and second-order dependencies are removed. The assumptions that apply to FA also apply to PCA. See Ref. (92) for a thorough resource on PCA.

In ICA, basis vectors give a result such that the resulting vector is an “independent component” of the original data. To perform ICA, we project the data on a basis vector and measure the kurtosis as a result. Then, we change the basis vector slightly and measure kurtosis again, typically through gradient ascent, until kurtosis is maximized. In fMRI time series, ICA is attractive relative to PCA and FA because it assumes that the data components are non-Gaussian signals and that raw data are noisy, non-stationary, and produced by several source signals, which are reasonable assumptions for fMRI (see Ref. (93) for theoretical background; see also Ref. (94) for an empirical test and suggestion that ICA in fMRI is related to the sparsity rather than independence of components *per se*).

We might select FA, PCA, or ICA when we are interested in a latent component representing the contributions of many voxels distributed across the brain. This represents an abstraction that extends beyond the multivariate techniques described above: in a component-based analysis, we assume that each component represents an important shared property of multi-voxel time series. Then, we can consider components as larger units of potential cognitive relevance by relating component spatial distributions or intensity against behavioral performance. This is especially relevant when we are interested in testing psychological constructs presumed to require contributions from multiple regions relatively near in time. For example, many working memory or executive functioning tasks require coordination between many regions, and a component-based analysis can allow the TBI researcher to identify and test larger systems’ relevance to TBI.

#### 4.2.4 Graph Theoretic Analyses

The multivariate time series analysis and decomposition techniques described above are all thought to represent or analyze temporal relationships between nodes in brain networks. Graph theoretic analysis is a specific approach to analyzing brain networks in which the brain network is represented in the mathematical object (“graph”). There are now many good reviews on graph theory in fMRI analysis, including the use and interpretation of network statistics in neuroimaging data (95), challenges (55), and progress (96) for graph theory in cognition, network analysis in nervous system disorders (97), and specifically TBI (98). The reader is encouraged to consult these primary references for specific content areas. Here, I briefly refer to major themes in graph theoretical analysis as relevant to contextualize its application to fMRI research in TBI.

In fMRI network analysis, the elements of the adjacency matrix *A* often include covariance, full correlation, partial correlation, coherence, and mutualized information. As with the multivariate time series techniques described above, the reliability and validity of these definitions of network edges remains an area of active research. After the adjacency matrix is specified, we can choose to either retain the edge weights, or binarize them according to a threshold. Once the fMRI adjacency matrix is defined, we can use concepts from graph theory to quantify network organization from the level of individual nodes and edges through the network as a whole. In fMRI networks, we can examine the intrinsic organization of the brain (that observed during rest and otherwise robust across many cognitive states) as well as its changes during cognitive tasks, in diseases, and over time scales from seconds to years.

In human functional brain networks, at a macro-scale level of organization, the brain exhibits a “small-world” topology that maintains an efficient balance between local and distributed information processing, via high clustering within network modules and short path lengths between them (99, 100). Brain network organization can be characterized to sit between three extremes of scale-free, regular, and random wiring characteristics (101). The scale-free component represents a high *degree* (number of connections to a node) diversity and strong hierarchical organization that includes highly connected “hub” nodes. Hubs can be identified by an unexpectedly high number of connections given all connections in the network at the level of the entire network, within specific modules, and between modules (102), and are thought to play key roles in regulating information processing across the network (102).

At the system level, fMRI studies demonstrate that the brain is organized into several major hierarchical intrinsic networks that can be observed during rest (103), activate with one another during tasks (53), and can be used to accurately identify individuals (104). These are often thought to represent systems with distinct cognitive roles, including fronto-parietal and cingulo-opercular control networks, dorsal and ventral attention networks, a salience network, a default mode network, primary somato-motor systems, and subcortical systems. The regions comprising these modules are often activated with one another in cognitive tasks, and functional connections across the brain robustly predict cognitive activations among these systems during various tasks, suggesting that these systems form basic building blocks in high-level cognitive organization (52).

At the node level, numerous measures have been defined that quantify the role of nodes in networks. In human neuroimaging data, each measure emphasizes complementary information about brain region roles. In addition to “hub” coefficients (102, 105), numerous measures have been developed to quantify the connectedness of nodes in the network (degree or strength in weighted networks), involvement in short paths across the network (betweenness centrality), connectedness with local neighbors (clustering coefficient or local efficiency), connectedness to important nodes in the network (eigenvector centrality), and interactions with multiple communities in the functional network (participation coefficient). At the edge-level, *edge* betweenness centrality quantifies the involvement of individual edges in short paths across the network. See Ref. (95) for a thorough discussion of commonly applied statistics and their interpretations. See Ref. (2) for a discussion of null network selection and community detection and summarization in brain networks.

We should select a graph theoretic analysis for our data when we are interested in making inferences about the organization of graphical features in TBI networks. Because mathematical concepts from graph theory are typically selected to reflect dissociable aspects of communication and information processing across networks, we can select specific measures for their potential theoretical link to behavior. Because other alternative analyses do not express these mathematical roles explicitly, such associations cannot be determined without a graph theoretical analysis. The unique value of these techniques to others in determining the basis of behavior in health in disease remains an open an increasingly active area of inquiry (55).

## 5 Findings in fMRI Research in TBI

There is now a substantial literature representing the use of fMRI to study TBI in varying degrees of severity, cognitive domains, and mechanisms of injury. Several excellent reviews of specific findings can be found elsewhere (98, 106–109). Here, I focus on several illustrative findings and themes concerning brain reorganization hypotheses following TBI. Despite heterogeneous injury presentations, cross-sectional designs in neuroimaging and TBI have proven informative, especially in contexts such as nearly ubiquitous working memory dysfunction following TBI (60, 67, 68). However, intensive approaches that focus on understanding reconfigurations at the level of individuals are necessary to confront the broader landscape of neuroplastic changes following TBI. For simplicity and general appeal, I review major themes addressing neuroplasticity hypotheses examined in cross-sectional studies and those that scale to entire brain networks. Later, in “Neuroplasticity in a naturally heterogeneous syndrome,” I discuss some approaches to understand neuroplasticity in the naturally heterogeneous context of TBI.

### 5.1 GLM Findings in TBI

GLM-based analyses have demonstrated that increased fronto-parietal BOLD signal amplitudes during working memory performance—a hallmark of post-TBI BOLD responses (60, 67)—decreases with sustained task performance and modulates with load similarly to healthy individuals. This suggests that bilateral fronto-parietal recruitment in TBI functions as a latent cognitive control mechanism that is differentially recruited in TBI (an increased mean BOLD signal) rather than a cognitive reassignment (68). Studies using continuous performance tests (110, 111), which require sustained cognitive control and task-set maintenance, and a study using a visuospatial attention task (112) reveal similar findings in cognitive control systems.

GLM studies in language processing demonstrate that functional recruitment of homotopic regions in the right hemisphere is observed post-TBI (113). In a study of young epileptics with left-lateralized lesions early in life, perilesional cognitive reassignment was observed when damage occurred in or near Broca’s area, and damage in regions remove from classical language areas was associated with non-left language lateralization in four out of five cases (114). In these language studies, BOLD response profiles are more consistent with cognitive reassignment and highlight the importance of examining the effects of the time-course of change following injury as well as indirect effects of lesions on processes.

### 5.2 Bridging from Regional fMRI Analyses to Functional Connectivity

Bridging BOLD signal amplitude analysis to functional connectivity, one study used a mixed-effects model to examine associations between elicited functional connectivity changes and BOLD variability in fronto-parietal regions during an n-back task (115). Right prefrontal cortex activation was positively associated with elicited connectivity within and between persons in health and TBI. In addition, right prefrontal cortex BOLD amplitude was positively associated with response times within and between subjects in each group, whereas right parietal activity was negatively related to response times in both groups. This indicates that right prefrontal cortex is an important upregulator of network connectivity in response to cognitive demand, whereas right parietal cortex activation is associated with better (faster) performance (115). These findings suggest that right prefrontal cortex and network connectivity as well as parietal cortex can serve as dissociable latent compensatory resources in TBI. Tasks that equilibrate working memory demands and vary cognitive domain may further test this hypothesis in TBI samples.

Extending from signal connections to multiple sets of connections using robust multivariate methods, some initial studies identify altered cognitively relevant connectivity in TBI. uSEM-based analyses have revealed reductions in right-hemispheric signaling and anterior–posterior shifting during working memory habituation (116) and better learning with increased fronto-parietal connectivity following severe TBI (117). In the former study, the effect of task on activity was robustly observed in the left parietal cortex in healthy group and in the right parietal cortex in the TBI group. In conjunction with GLM-based studies (68), uSEM-based studies suggest that the reliance on frontal control mechanisms subsides with task familiarity, and that brain networks settle into consolidated fronto-parietal interactions in both health and TBI. In the case of working memory following brain injury, it appears that fronto-parietal latent resources are transiently recruited and detectable in both BOLD amplitude and functional connectivity.

### 5.3 Scaling to Graph Theoretic Analyses

**Figure 6:**
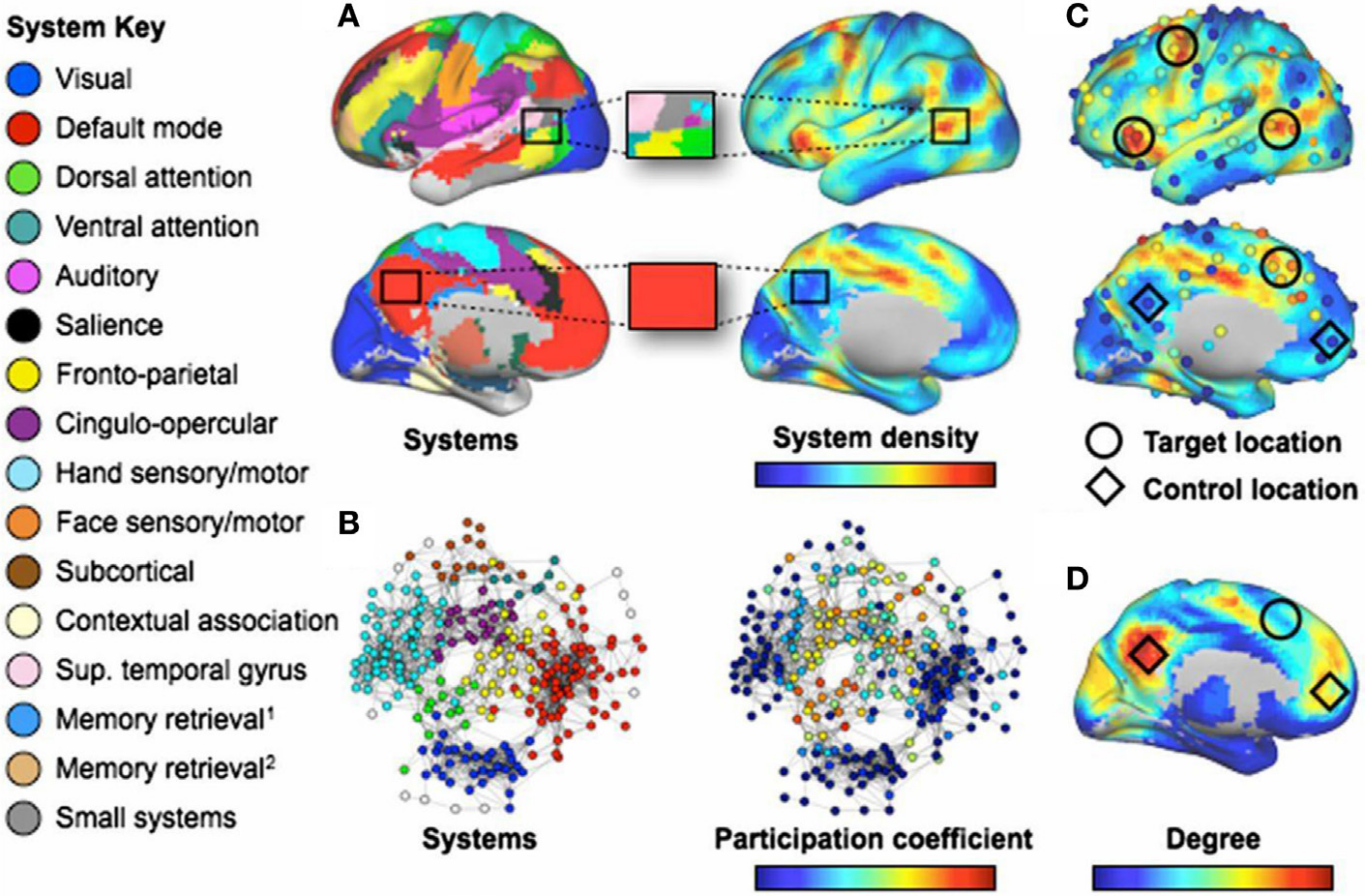
Regions involved in many systems produce the broadest deficits in stroke. [**(A)**, Left] Consensus communities (systems) from Ref. (103, 122). *(Right)* Plot of the density of these systems across the cortex. The blowup boxes illustrate how certain cortical locations contain many systems (Upper: a target location) or few systems (Lower: a control location). [**(B)**, Left] Communities (systems) in a brain-wide network. *(Right)* Node participation coefficients, with warmer colors denoting nodes that display spontaneous BOLD activity that correlates with multiple systems. **(C)** Plots of system density and participation coefficients overlaid. Target locations (circles) show where lesions produce multi-system impairment. Control locations (diamonds) do not produce widespread impairment. Only four of six target locations are shown (those in the left hemisphere). **(D)** Node degree, with warmer colors indicating higher degree. Note that regions with high degree may have low system density and/or participation coefficient *(C)*. The names for systems given in the left-side legend are either those commonly used (e.g., DMN) or are demonstrated functional properties of the labeled system when no consensus name exists. Figure and caption modified and reproduced with permission from Ref. (121).

Graph theoretic studies typically focus on the topological organization of connectivity involving many regions at once. In TBI, fMRI networks reveal increased connectivity degree and strength as well as reduced efficiency. Disturbances involving increased or decreased functional connectivity can be found across all macro-scale functional networks (118). Over the first several months of recovery, “hyperconnectivity” decreases in resting fMRI networks in individuals with TBI during a critical period of cognitive recovery (119); however, the relationship between connectivity changes and cognition was not examined across subjects. Importantly, if the so-called “salience” network’s suppression of the default mode network is disrupted via damage to a tract connecting the right anterior insula to the midline presupplementary motor area, failures in behavioral inhibition are observed (120). Failure to suppress the default mode network may more generally lead to interference in normal network interactions across the brain (112). More recently, it was discovered that the effects of focal brain lesions on behavior depends on their topological network location. Damage to regions with a high functional participation coefficient and “system density” result in widespread cognitive deficits in many domains, whereas local network hubs produce more circumscribed deficits (121) (see Figure 6).

Considered together, these findings establish important foundations for TBI research involving classical cognitive regional roles, network-defined roles, and the nature of reorganization and vulnerability in the human brain. Cognitive control systems and homotopic regions may serve as latent resources and sites of cognitive reallocation that are heavily relied upon during cognition following TBI in the context of network compromise. Specific damage to pathways that affect communication between systems that respond to relevant external stimuli (the salience network) and the brain’s default mode network can selectively disrupt inhibition and the suppression of internally focused attention. In extreme cases, damage to key nodes in the system lead to catastrophic consequences for cognitive function. This suggests that latent resources may be conditionally recruited as a function of the degree of global decline, which is predictable based on the participation coefficient of damaged nodes in the network. Specifically, latent resources and reallocation may only be likely to activate if the network has a certain degree of intact global system organization supported by key nodes. Future analyses could examine control system and homotopics recruitment in the context of varied control system damage to test this hypothesis.

#### 5.3.1 Conceptual Caveats

Some caveats to the use of the aforementioned approaches in TBI apply. Conceptually, *functional* BOLD amplitudes, patterns, connectivity, and networks should not be confused to be synonymous with their *cognitive* and *neural* analogs. Indeed, some have criticized BOLD-based connectivity measures as fundamentally low-dimensional and limited in their ability to represent brain interactions (123). Even if the mapping between neural and hemodynamic states was entirely understood, the BOLD signal would offer a useful but limited lens through which to examine cognitive function. While the framework advocated in this article may aid us in clarifying hypotheses and inferences about changes in BOLD signal patterns following TBI, we should be cautious to use language clearly and not oversell findings based on the technique. We should supplement the use of BOLD fMRI with the numerous and increasing techniques available to the cognitive neuroscience researcher. We should also seek to reconcile questions about brain reorganization with core theories of cognitive function, localization, and distribution in the brain.

## 6 The Future of fMRI Analysis in TBI

There remain several open frontiers in fMRI analysis in TBI. I discuss possibilities for multimodal imaging analyses and the importance of model-based predictive frameworks that may move use beyond associative studies to a formal predictive science for fMRI analysis in TBI.

### 6.1 Multimodal Analysis

Given the hemodynamic dilemma and spatiotemporal limitations to fMRI, using additional techniques may be helpful to understand TBI responses to brain injury. This is especially important when attempting to explain a significant percentage of cognitive-behavioral variability and outcomes in TBI. Due to the limits inherent in fMRI in terms of intersubject variability in the fundamental hemodynamic response function, potential hemodynamic alterations in TBI, and lack of a “ground truth” recording of neural states, we can expect that many observed associations could be small and unreliable. This should encourage us to apply multiple functional techniques in to assess different domains of spatiotemporal inquiry and consider opportunities for multi-approach integration.

One promising area for multimodal analysis may involve integrating anatomical and functional data to predict BOLD changes. At a high level of organization, diffusion imaging-derived anatomical networks predict resting and task-based functional connectivity (124, 125). Simulated damage to the brain’s anatomical connectivity profile results in changes in simulated resting connectivity, with the greatest consequences emerging from lesions to the cortical midline and temporo-parietal junction (126). How anatomical network damage leads to the expression of both behavioral symptomatology and compensation or reallocation-relevant functional topology changes following TBI is an open area.

One line of evidence has begun to emerge that suggests how to link anatomical and functional imaging data. In healthy development, diffusion imaging-based connectivity “fingerprints” can predict BOLD pattern development in the visual word form area, suggesting that underlying anatomy can determine the location of specific cognitive processes as neuroplastic processes unfold (127). If a similar anatomical guiding principle underlies functional neuroplasticity following TBI, it may be possible to predict reorganized activity using diffusion imaging data in cognitive and motor domains. Beyond fMRI, similar investigations could be conducted using EEG (128), MEG (129), or electrocorticography (130) to define functional response profiles and attempt to clarify the nature of the BOLD signal (38) and how anatomy guides neuroplastic changes across spatiotemporal scales.

### 6.2 From Association to Predictive Models

An important goal in clinical neuroimaging is to predict disease incidence, recovery trajectories, and the effects of intervention. As a term, “prediction” is loosely applied in fMRI research applied to TBI. For example, some work uses control subject-based network measures to predict cognitive performance in TBI samples (121) or correlation-based analyses to associate connectivity patterns with cognitive measures (131). However, this is not the same as developing a model that is sensitive and specific to a prototypical pattern of behavioral changes and that can predict them ahead of time for a single person. This agenda will likely require a careful combination of biologically validated information paired with robust behavioral measurements. For example, some emerging work in comparative models indicates that BOLD response recovery in the first 56 days following injury is associated with functional motor recovery independently from cortical lesion volume or thalamic neurodegeneration but associated with preserved myelinated fibers in layer VI of region S1 (132). This suggests that basic BOLD responses may have some basis in white matter preservation and be sensitive to some aspect of biology that is responsible for functional outcomes.

Given the aforementioned limitations to fMRI, it is improbable that the most effective predictions for variable behavioral outcomes in TBI will be based solely on this technique. It is likely that robust predictive models will involve a combination of anatomical, functional, demographic, and cognitive-behavioral information. Indeed, different types and combinations of data may predict different cognitive-behavioral profiles after brain injury (133). As data increase in precision, number of modalities, and number of subjects across academic research centers, the “big data” era (134) offers several opportunities to develop such models (135). Machine learning is influencing neuroscience research broadly and offers the ability to generate powerful predictive capabilities. The challenges for prediction in TBI will involve practical algorithms that are easy to implement in clinical contexts and robustly cross-validated in independent samples. Ideally, as our current focus on high-dimensional multimodal data matures, we will be able to identify simpler principles and new techniques that minimize costs with optimal predictive gains.

In addition, there now exist several robust cognitive theoretical models (“architectures”) that predict many clinically measured behaviors with high fidelity. Some of these models are specific to cognitive control (136), a process that is emerging as a quintessential yet limited (137) latent compensatory resource following TBI (60, 68). Cognitive control can be thought to be based in a distributed system of brain nodes with distinct computational roles. One account suggests that the anterior cingulate cortex modulates cognitive control by computing its expected value during cognitive processes (138). If we can identify evidence that the value of control differs in TBI versus healthy controls, it is possible that this effect is mediated by the anterior cingulate and its association with cognitive control networks. Efforts in computational neurology associating theoretical predictions from cognitive models with brain measures are proving fruitful across computational neuroscience, and its extension to clinical neuroscience may accelerate progress in TBI research.

### 6.3 Neuroplasticity in a Naturally Heterogeneous Syndrome

Earlier, I reviewed a basis for clarifying the nature of “brain reorganization” in TBI to avoid circular reasoning or semantic ambiguity. Then, I briefly reviewed literature that applied similar reasoning to test hypotheses about functional neuroplasticity measured with fMRI in cross-sectional designs. In that context, cross-sectional studies investigated commonly observed working memory dysfunction due to TBI, supporting productive inferences discriminating between competing accounts of neuroplasticity. However, while cross-sectional designs are appealing for some hypotheses for simplicity and to maximize power, they may be inadequate when both heterogenous anatomical damage and symptom categories are observed. I make two observations about this challenging frontier. First, we can apply the same dismantling logic described above at the level of the individual with some methodological extensions given appropriate reference comparisons. Second, with increasingly large-scale datasets and data sharing capabilities, we could leverage resources to perform larger scale hypothesis testing and personalized phenotyping for neuroplastic responses following TBI.

In principle, the general approach to understanding BOLD signatures of neuroplasticity described above could be applied to intensive within-subjects designs. However, validating relationships at the level of single individuals is necessary to identify whether BOLD–behavior relationships represent uniform neuroplastic responses across individuals, or merely measures sensitive to central tendency in the context of heterogeneous responses. Indeed, demonstrating that psychophysiological processes are uniformly observed within individuals over time is an important requirement to validate observations at the group level (139, 140). For example, demonstrating that trial-level performance within every individual in a post-injury sample exhibits similar BOLD responses to cross-sectional results would be compelling evidence that uniform neuroplastic responses have occurred after heterogeneous TBIs. To test for such consistency, we could map individual-level BOLD–behavior relationships and compare their locations, effect sizes, and reliability across individuals. This would allow us to determine whether central tendencies observed in cross-sectional designs are representative of common sites of neuroplasticity in the context of pathological heterogeneity. In addition, though not commonly used in fMRI studies, matched case–control study designs (141, 142) with adequate bias control (143) could potentially help to elucidate subject-specific behavior–BOLD response relationships within a particular subject compared to individually matched healthy subjects.

Finally, several large-scale efforts could facilitate research focused on common *versus* individual neuroplastic effects. These studies can help us address a fundamental limitation in TBI research: it is unethical to purposefully cause a TBI in healthy individuals. Thus, while some *post hoc* information can be obtained after TBI, study designs that allow us to make pre- and post-injury comparisons fill a critical knowledge gap. Projects that collect data in normative groups and follow them for years in development from youth to adulthood (144) and late aging (145) will incidentally include some individuals that suffer TBI, facilitating true within-person cognitive dismantling designs. Importantly, sufficiently large longitudinal and cross-sectional studies can facilitate Bayesian approaches to identify cognitively relevant brain activation as has recently been explored in large healthy datasets (146, 147). With some modifications, this could allow us to detect that a certain cognitive process has been reallocated by detecting whether novel BOLD activity in an individual with TBI conforms to a prototype observed in a much larger repository. Conversely, this would also allow us to identify whether activity observed following TBI can be identified as a reconfiguration of a healthy pattern *versus* a truly novel pattern that requires further person-specific study. These larger scale efforts could be supported by large multi-PI funded protocols and increased open data sharing across collaborators.

## 7 Conclusion

The framework discussed here is one way to organize the numerous cognitive questions and techniques used in TBI research. If cognitive reallocation and latent compensatory mechanisms encompass all of the basic neurocognitive responses to TBI, careful experimental design and an open mind to their likely complex expression in BOLD responses will result in fruitful fMRI research. More likely, nature will surprise us, and we will need to adjust our frameworks accordingly. Many well-designed cross-sectional, longitudinal, and case–control studies will be needed to confront this challenge. However, these studies will only be as valuable as their ability to discriminate among competing accounts of neuroplastic changes following TBI. Given the tremendous computational, analytic, and theoretical resources available to us, fMRI may have much yet to reveal about the nature of cognitive neuroplasticity following TBI.

## Author Contributions

The author confirms being the sole contributor of this work and approved it for publication.

## Conflict of Interest Statement

The author declares that the research was conducted in the absence of any commercial or financial relationships that could be construed as a potential conflict of interest.

## Footnotes

^1^Unfortunately, this term is frequently used very loosely in cognitive and clinical literatures. The reader should consult Ref. (61) for a rigorous treatment of the issue and the stakes for science at large.

^2^While outside the scope of this perspective, “multiple realizability” may be a challenge for this approach in principle (62).
